# Novel All‐Nitrogen Molecular Crystals of Aromatic N_10_

**DOI:** 10.1002/advs.201902320

**Published:** 2020-03-30

**Authors:** Shijie Liu, Lei Zhao, Mingguang Yao, Maosheng Miao, Bingbing Liu

**Affiliations:** ^1^ State Key Laboratory of Superhard Materials Jilin University Changchun 130012 China; ^2^ School of Physics and Engineering and Henan Key Laboratory of Photoelectric Energy Storage Materials and Applications Henan University of Science and Technology Luoyang 471003 China; ^3^ School of Optoelectronic Science and Engineering University of Electronic Science and Technology of China (UESTC) Chengdu 610054 P. R. China; ^4^ Department of Chemistry and Biochemistry California State University‐Northridge Northridge California 91330 USA; ^5^ Beijing Computational Science Research Center Beijing 10084 China

**Keywords:** high energy density materials, molecular crystals, N_10_ molecule, polynitrogen

## Abstract

Nitrogen has unique bonding ability to form single, double, and triple bonds, similar to that of carbon. However, a molecular crystal formed by an aromatic polynitrogen similar to a carbon system has not been found yet. Herein, a new form of stable all‐nitrogen molecular crystals consisting of only bispentazole N_10_ molecules with exceedingly high energy density is predicted. The crystal structures and the conformation of N_10_ molecules are strongly correlated, both depending on the applied external pressure. These molecular crystals can be recovered upon the release of the pressure. The first‐principles molecular dynamics simulations reveal that these all‐nitrogen materials decompose at temperatures much higher than room temperature. The decompositions always start from breaking off N_2_ molecules from the nitrogen ring and can release a large amount of energy. These new polynitrogens are aromatic and are more stable than all the other polynitrogen crystals reported previously, providing a new green strategy to get all‐nitrogen, nonpolluting high energy density materials without introducing any metal or other guest stabilizer.

## Graphical abstract

A new form of all‐nitrogen molecular crystals is predicted, which consists of only N_10_ molecules with exceedingly high energy density. These new structures are aromatic and more stable than all the other polynitrogen crystals reported previously, providing a new green strategy to get all‐nitrogen, nonpolluting high energy density materials without introducing any metal or other guest stabilizer.

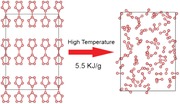

## 1 Introduction

In the periodic table, carbon and nitrogen are neighbors and both possess unique bonding ability to form single, double, and triple bonds, which makes them possible to form diverse molecular crystals. In fact, carbon does form rich molecular crystals, such as the fascinating family of fullerenes composed of aromatic six‐ membered and five‐membered rings.^[^ ^1^, ^2^, ^3^ ^]^ Nitrogen is abundant in the earth and can only form molecular crystals of N_2_ at low temperatures. Recently, the theory predicts that pure nitrogen can form chain‐like N_8_ and N_6_ molecular crystals.^[^ ^4^, ^5^ ^]^ However, a molecular crystal formed by an aromatic polynitrogen molecule similar to a carbon system has not been found in a full‐nitrogen system, which is a great fundamental interest.

An important goal of obtaining such a molecular crystal is that the material can be used as a green, nonpolluting, high energy density material (HEDM), which not only has a very high energy density about five times that of 2,4,6‐trinitrotoluene (TNT),^[^ ^6^ ^]^ but also has a decomposition product of only N_2_. In fact, an aromatic pentazolate (N_5_) structure in organic compounds has been reported as early as the 1900s.^[^ ^7^ ^]^ In 2002, N_5_^−^ was produced in the gas phase by cleaving the C—N bond in the substituted phenylthiazole.^[^ ^8^ ^]^ Recently, a series of solid compounds consisting of cyclic N_5_^−^ were synthesized under high pressure, such as CeN_5_ and LiN_5_.^[^ ^9^, ^10^ ^]^ In 2017, a breakthrough was made by successfully synthesizing this cyclic N_5_^−^ in five metal pentazolate hydrate complexes at ambient condition.^[^ ^11^, ^12^ ^]^ Since cyclic N_5_^−^ anion is aromatic, it has excellent stability and can be stabilized even at 100 °C. These findings in cyclo‐N_5_ anion open the door to the fascinating pentazole chemistry. However, the aromatic molecular crystal formed by the pure cyclic N_5_ has not been reported so far.

In addition, the study of polymeric nitrogen under high pressure has also attracted great attention. Since the first polymeric nitrogen structure was proposed in 1985,^[^ ^13^ ^]^ a series of new structures have been designed, such as chain‐like,^[^ ^14^, ^15^, ^16^, ^17^ ^]^ layer,^[^ ^18^, ^19^ ^]^ and 3D structures.^[^ ^20^, ^21^, ^22^ ^]^ Experimentally, a breakthrough was made in 2004: Eremets et al.^[^ ^6^ ^]^ observed a cg‐N structure from 110 to 140 GPa above 2000 K. Subsequently, Tomasino et al.^[^ ^23^ ^]^ synthesized a new type of polymeric nitrogen called LP‐N at 126–175 GPa by direct laser heating of N_2_ in 2014. Recently, Laniel et al.^[^ ^24^ ^]^ synthesized a layered polymeric nitrogen structure with a hexagonal structure at about 250 GPa. However, these polymeric nitrogen structures are atomic or layered structures, and no aromatic molecular crystals formed by pure nitrogen have been found. Therefore, we still need to study whether there are aromatic molecular crystals in the nitrogen system.

Here, we predict a new form of aromatic polynitrogen crystals composed of all nitrogen N_10_ molecules from theoretical simulations. The N_10_ molecules are held together by van der Waals (vdW) interaction to form the molecular crystal and each molecule can be taken as a bispentazole molecule. This new structure has excellent stability and is metastable at ambient conditions, which is superior in energy to the previously proposed N_8_ and N_6_ molecular crystals. The crystal is a potential HEDM due to the high energetic N_10_ polynitrogen molecules. The prediction of such ring‐like polynitrogen stable at ambient pressure may open up a new area of nitrogen chemistry to search for full‐nitrogen aromatic molecular crystal and also guide the synthesis of new polymeric nitrogen.

## 2 Results and Discussion

**Figure 1:**
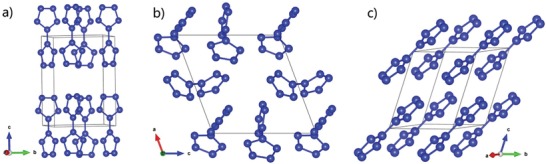
a–c) Energetically favorable structures of V phase, I phase, and P phase, respectively, according to the molecular configurations of the building blocks.

Our structural search of nitrogen crystals was performed in the pressure range of 0–100 GPa by using simulation cells containing up to 40 N atoms. A new form of aromatic crystal structures composed of N_10_ molecules with space groups of P4/mbn (*Z* = 2), P1 (*Z* = 3), and P‐1 (*Z* = 1), respectively, has been obtained and all could be stable at ambient pressure. We have listed the optimized lattice parameters of three structures in Table S1 (Supporting Information). Since the N_10_ molecule has two N_5_ rings, we named the molecule as bispentazole. Their structures are shown in **Figure** **1**. Examination revealed that the P4/mbn structure consisting of two identical bispentazole molecules is the most stable phase at ambient pressure (see next), in which the bispentazole molecule with a D2d symmetry is formed by two pentazole rings (N_5_) perpendicular to each other. The bond lengths and bond energy of bispentazole molecule are shown in Tables S2 and S3 (Supporting Information). The configurations of the bispentazole molecules in the other two crystals are different, i.e., the angle between the two N_5_ rings in the bispentazole molecule is between 70° and 90° in the P1 structure and is 0° in the P‐1 structure (i.e., the two N_5_ rings are parallel to each other). This indicates that the molecular configurations have important influence on the crystal structure and the stability of the three phases. According to these features in the corresponding molecular configurations, these three structures are named as V phase (vertical phase), I phase (inclined phase), P phase (parallel phase), respectively. Bader analysis shows that each bispentazole molecule in these structures is electrically neutral, suggesting only a weak van der Waals interaction exists between them. In addition, we take the V phase as an example to calculate the cohesive energy of the molecular crystals of N_10_. The calculated results show that the cohesive energy of the crystal of the molecule is 48.8 kJ mol^−1^ (11.6 kcal mol^−1^). This cohesive energy is significantly higher than the cohesive energy of α‐phase of N_2_ due to the strong electrostatic interaction between the N_10_ molecules, similar to the previously reported N_8_ molecules^[^ ^4^ ^]^ as well as CO_2_^[^ ^25^ ^]^ and acetonitrile.^[^ ^26^ ^]^ Moreover, we calculate the cohesive energy without vdW interaction, which is 10.0 kJ mol^−1^ (2.4 kcal mol^−1^). It is clearly shown that the cohesive energy of the N_10_ molecular crystal with vdW interaction is increased by almost four times than that without vdW interaction. This result indicates that vdW interaction plays an important role in the stability of the system.

**Figure 2:**
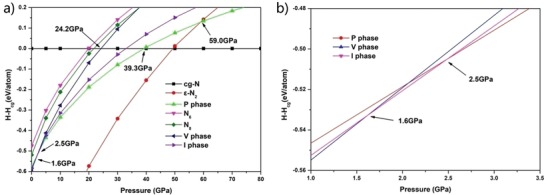
a) The enthalpies difference curve (relative to cg‐N structure) of three bispentazole molecular crystal structures compared to other structures; b) a magnified view of the enthalpies difference curve with the pressure range of 1–3.5 GPa.

The enthalpies of the three molecular crystal structures together with other known structures are plotted as a function of pressure in **Figure** **2**. We introduced the van der Waals interaction by using the DFT‐D2 method. The results show that our three structures have much lower energy at low pressure compared to the well‐studied cg‐N. At ambient pressure, among the three newly proposed structures, the energy of V phase is the lowest and P phase is the highest, while I phase in the middle. As pressure gradually increases, I phase structure becomes the most stable with the lowest energy above 1.6 GPa; at pressure higher than 2.5 GPa, the energy of the P phase becomes the lowest, as shown in Figure 2b. It is worth noting that all these three crystals have lower energy than cg‐N at pressure below 39.3 GPa, and lower energy than ε‐N_2_ above 59.0 GPa. Moreover, the three crystal structures also exhibit lower energy over the entire pressure range than the previously reported N_8_^4^ and N_6_^5^ molecular crystals. Note that both cg‐N and N_8_ structures have been successfully synthesized in experiment, while N_5_ ring has also been recently identified in cyclo‐N_5_‐based complexes. Therefore, it is very promising to synthesize the N_10_ molecular crystals proposed here in experiment. We also calculated the X‐ray diffraction patterns and vibrational spectra of the V phase for future experimental reference (Figures S1 and S2, Supporting Information).

Cyclic N_5_^−^ is a potential candidate for full‐nitrogen energetic materials and can be isolated only from acidic solution. Recent researches^[^ ^27^, ^28^ ^]^ reported the stability and energy storage mechanism of the cyclic N_5_^−^ in acidic solution, namely, the resultant force of the circumferential H↔H repulsion stretches the N:H—O bond to lengthen the N:H and shortens the H—O, which weakens the N:↔:N repulsion and shortens the N—N bond along the cyclo‐N_5_^−^, thereby making the system store energy; the N:↔:N repulsion destroys or reduces the aromaticity of cyclo‐N_5_^−^ and makes cyclo‐N_5_^−^ unstable, while the strong acidic solution reconstructs or enhances the aromaticity of cyclo‐N_5_^−^ and stabilizes cyclo‐N_5_^−^, making cyclo‐N_5_^−^ stable only in sufficiently acidic conditions. Similar to the effect of H↔H repulsion stretching (acidic solution) in references, the pressure here plays a significant role for both energy storage and stability of N_10_ molecular crystal. In order to further analyze the stability and energy storage mechanism of N_10_ molecular crystal, we take the V phase as an example for the following discussion. As shown in Figure S3 (Supporting Information), we show the orientation of each N_10_ molecule in the V phase. We calculate the Shannon aromaticity (SA) that is used to characterize the aromaticity, the distance between N_10_ molecules and the bond length within N_10_ molecules under different pressures, as shown in Table S3 (Supporting Information). By comparing the SA under different pressure, we find that with the increasing of pressure, the aromaticity of N_10_ molecule increases gradually increased with the decrease of SA value, which also shows that the stability of N_10_ molecules is gradually increased. By comparing the intermolecular distances and the intramolecular bond lengths under different pressure, we find that the intermolecular distances of N_10_ molecules and lengths of N—N bond in N_10_ molecule appear to shorten gradually with the increase of pressure, that is, with the increase of the intermolecular repulsion force, the lone pairs of electrons extend radially away from each molecule, weakening the circumferential repulsion between the lone pair of electrons and shortening all N—N bonds in the N_10_ molecule, and thus enhance all N—N bonds by ≈26.3 kcal mol^−1^ at 25.0 GPa. Therefore, high pressure plays a significant role for both energy storage and stability of N_10_ molecular crystal.

**Figure 3:**
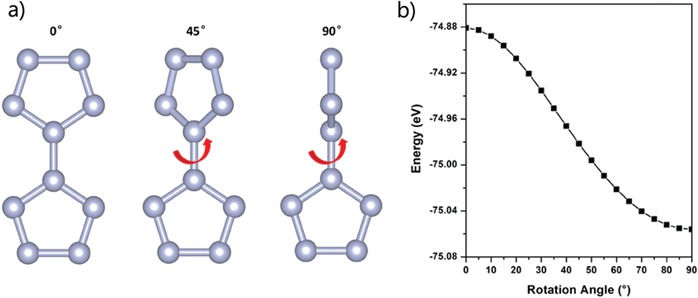
a) N_10_ molecule configurations with different rotation angles. b) Total energies as a function of N_5_ orientation at ambient pressure of N_10_.

To understand the configuration‐dependent systematic energy variation of the three structures, we calculate the energy of a bispentazole molecule (i.e., building block) with varying the angle between the two N_5_ rings in the molecule (**Figure** **3**a). The results are shown in Figure 3b. It can be seen that at ambient pressure, the bispentazole molecule with the two N_5_ rings parallel to each other (P‐configuration) has the highest systematic energy; its energy gradually decreases as the angle increases (I‐configuration) and reaches the lowest energy when the two N_5_ rings are perpendicular to each other (V‐configuration). This is also consistent with previous literatures,^[^ ^29^ ^]^ confirming that the vertical bispentazole molecule has the lowest energy in all N_10_ isomers. This also explains why V phase has the lowest energy at ambient pressure and P phase has the highest energy. In addition, as the pressure increases, the structural transitions from V phase to I phase and then to P phase can also be explained by the molecular configurations in the three phases, i.e., at ambient pressure, the bispentazole molecule with V‐configuration needs to occupy the largest volume and possess the lowest energy; with pressure increasing, the bispentazole molecule tends to have V‐configurations, causing the increase of the structural energy; at higher pressure, the bispentazole molecule forms P‐configuration and the systematic energy is increased to the maximum.

**Figure 4:**
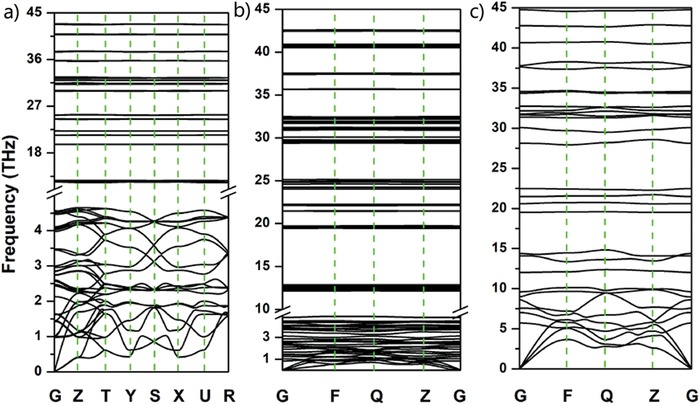
a–c) Phonon spectrum of V phase at 0 GPa, I phase at 2 GPa, and P phase at 20 GPa, respectively.

Furthermore, we calculated the phonon spectra of V phase at 0 GPa, I phase at 2 GPa, and P phase at 20 GPa, respectively, to confirm the dynamic stability of their molecular crystals. The results are shown in **Figure** **4**. It can be seen that all the three structures have no imaginary frequency under the corresponding pressure, indicating that these structures are dynamically stable. It is worthy to mention that no imaginary frequency is found in the phonon spectra of I phase and P phase at 0 GPa, suggesting that V, I, and P phases all can be metastable at ambient pressure.

**Figure 5:**
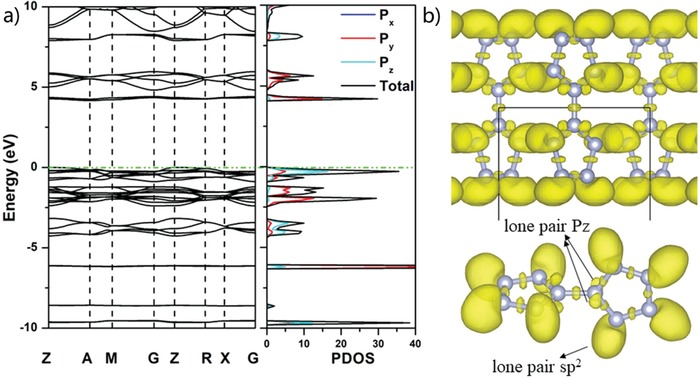
a) Band structure and PDOS; b) The calculated ELF isosurface of V phase at ELF = 0.83.

The electronic properties of the bispentazole molecular crystals have been further calculated by using the Perdew, Burke, and Ernzerhof (PBE) and the Heyd, Scuseria, and Ernzerhof (HSE) method, including the band structure, the partial densities of states (PDOS), and the electronic local functions (ELFs), as shown in **Figure** **5**. Take the V phase as an example, our calculation shows that it is an insulator with an indirect bandgap of 4.2 eV at the PBE level. Note that it is commonly accepted that the HSE method is one of the most accurate methods for band structure calculation while the PBE method usually underestimates the bandgap of the structure. Therefore, we have recalculated the band structure of the V phase, and the bandgap at 0 GPa is 6.1 eV at the HSE level (Figure S4, Supporting Information). Analysis of ELFs and partial densities of states suggests that the N atoms in N_5_ rings are sp^2^ hybridized with lone pair lobes pointing at the edge of the N_5_ ring, whereas the bridge N atoms bonding two N_5_ rings are sp^2^ hybridization with lone pair p_z_ at both sides of the bridge N. In each N_5_ ring of the N_10_ molecule, the bridge nitrogen atom provides two p_z_ orbits, and the other nitrogen atoms provide one p_z_ orbit, and a total of six electrons form a delocalized π bond. Therefore, the N_10_ molecule retains the aromaticity, which also makes it have lower energy and higher stability. In addition, our calculation of the electronic properties of the I and P phases also suggest their insulating features, and the bonding type in the corresponding bispentazole molecules is the same as that of V phase (Figures S5 and S6, Supporting Information).

**Figure 6:**
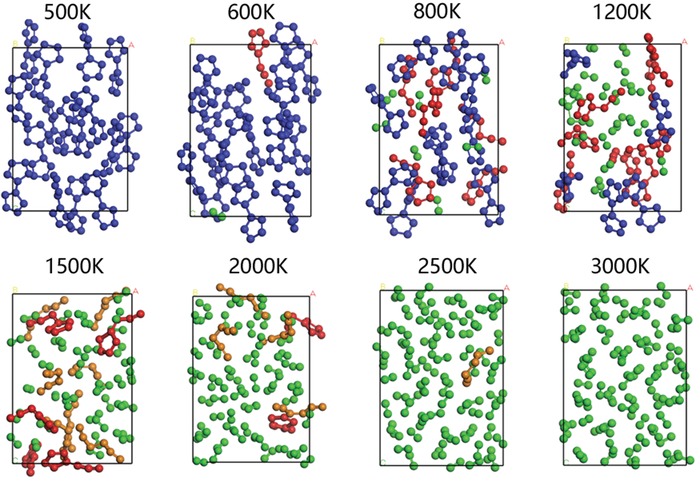
The snapshots at 500, 600, 800, 1200, 1500, 2000, 2500, and 3000 K after 20 ps in the MD simulations.

The thermal stability of V phase has been evaluated by using a supercell with 160 nitrogen atoms as a model, which is carried out by ab initio molecular dynamics (AIMD) simulations from 300 to 3000 K with a step size of 100 K. Snapshots of V phase structure taken at the end of 20 ps simulations are presented in **Figure** **6**. The framework of each bispentazolate molecule is stable up to 500 K after 20 ps dynamical simulation. As the temperature increases about 600 K, one of N_5_ rings in the bispentazolate molecule is opened and the bispentazolate molecule is decomposed into azidopentazole (N_8_) and N_2_ molecules. Note that the decomposed N_8_ can be taken as a molecule formed by bonding a N_5_ to an azide‐like N_3_ ion. It also indicates that the bispentazolate should have enough kinetic energy to cross the barrier and turn to be corrupt at the temperatures between 500 and 600 K. As the temperature increases, more bispentazolate molecules decompose into N_8_ and N_2_, and they completely decompose above 1500 K. Further increasing the temperature leads to the decomposition of the generated N_8_ into N_6_ and N_2_ molecules, in which the generated N_6_ molecule has a chain‐like configuration identical to that reported in previous literature.^[^ ^5^ ^]^ At temperatures above 2600 K, the decomposition product is only N_2_ molecules. Furthermore, we investigate the decomposition pathway of this N_10_ molecule by using climbing image nudged‐elastic band (CI‐NEB) method. Our calculations show that the transition barriers of the three decomposition processes are 10.1 kcal mol^−1^ for TS1 [N_10_→TS1→N_8_+N_2_], 12.0 kcal mol^−1^ for TS2 [N_8_→TS2→N_6_+N_2_], and 19.4 kcal mol^−1^ for TS3 [N_6_→TS3→3N_2_] (Figure S7, Supporting Information). The calculated results show that TS1 has a smaller reaction energy barrier, and TS2 and TS3 have relatively larger reaction energy barriers, which correspond to the lower decomposition temperature of N_10_ and the higher decomposition temperature of N_8_ and N_6_ in AIMD simulations. In addition, decomposition of N_10_ molecules into N_2_ molecules can release energy of 192.0 kcal mol^−1^ during this process.

The total energy calculation shows that V phase can release energy of 15.8 eV/cell under ambient pressure, corresponding to an energy density of about 5.5 kJ g^−1^. This is much higher than those typical energetic materials, such as TATB, RDX, and HMX, which have energy densities around 1–3 kJ g^−1^.^[^ ^30^ ^]^ In addition, due to the instability of a single cyclic N_5_ ring, nonenergetic ions are required to stabilize them in the complexes in previous works,^[^ ^9^, ^10^, ^11^, ^12^, ^31^, ^32^ ^]^ which reduces the energy density of the materials. In our predicted three polynitrogen crystals, the covalent bond between the two cyclic N_5_ rings naturally stabilize each other and thus form stable bispentazole molecular crystals even at ambient pressure. Since no impurity ions are introduced in our systems, this class of all‐polynitrogen crystal has a higher energy density and thus promises for a high energy density material. Our results also indicate that, compared with the polynitrogen system with chain‐like configuration, such as N_8_ and N_6_, ring‐like aromatic polynitrogen might be more stable building blocks for nitrogen allotropes in some structures that can be stable at ambient pressure, as the well‐known C_60_ in carbon allotropes. The existence of stable nitrogen crystals composed of all‐cyclic N_5_ may also guide the synthesis of a fully cyclic N_5_ system in experiment.

In summary, we have predicted a form of new all‐nitrogen aromatic molecular crystals, named as V, I, P phase, which are a type of N_10_ molecule formed by two five‐membered cyclic configuration units bonded to each other. The calculated phonon spectra indicate that crystal structures are metastable under ambient pressure, indicating that these structures have the possibility of synthesis in experiment. First‐principles molecular dynamics calculations show that the structures can exist stably at room temperature, and even to 500 K. The bispentazole molecule proposed here makes two N_5_ polynitrogen rings stable without introducing any impurity ions, which contains a high energy density and is therefore a potential high energy density material. These structures represent one possible way to synthesize a full‐nitrogen aromatic molecular crystal.

## 3 Experimental Section

The CALYPSO code^[^ ^33^, ^34^ ^]^ was used for structural search, and the underlying structural relaxation by using the Vienna Ab initio Simulation Package (VASP).^[^ ^35^ ^]^ The projector augmented wave (PAW) method to represent ion–electron interactions and the generalized gradient approximation (GGA) of the form proposed by PBE^[^ ^36^ ^]^ as the electron exchange correlation functional were used. In order to achieve excellent convergence of the total energy, the cutoff value of the plane wave was set to 900 eV. The Monkhorst–Pack method was used to sample the Brillouin zone for geometric optimization and self‐consistent calculations. To determine the kinetic stability of the molecular crystal structure, the PHONOPY code^[^ ^37^ ^]^ is used for phonon dispersion analysis. An AIMD simulation of 20 ps was performed to evaluate the thermal stability of all molecular crystals. CI‐NEB method^[^ ^38^, ^39^ ^]^ within VASP was used to find the minimum energy paths and the transition states for diffusion of N_10_ molecular. Covalent bond energies were calculated by the integrated value of crystal orbital Hamilton population (COHP) at the band energy using the HASEM package.^[^ ^40^, ^41^ ^]^

## Conflict of Interest

The authors declare no conflict of interest.

## Supporting information

Supporting Information
