# Supplementary material for: Novel All‐Nitrogen Molecular Crystals of Aromatic N10
Source: Adv Sci (Weinh). 2020 Mar 30;7(10):1902320. doi: 10.1002/advs.201902320 (PMC7237857; doi:10.1002/advs.201902320)
Supplement: Supplementary file 1 — Supporting Information [file ADVS-7-1902320-s001.pdf]

Copyright WILEY-VCH Verlag GmbH & Co. KGaA, 69469 Weinheim, Germany, 2020.

ADVANCED SCIENCE
Open Access

Supporting Information

for *Adv. Sci.,* DOI: 10.1002/advs.201902320

Novel All-Nitrogen Molecular Crystals of Aromatic N_{10}

Shijie Liu, Lei Zhao, Mingguang Yao, Maosheng Miao, and Bingbing Liu**

Supporting Information

Novel all-nitrogen molecular crystals of aromatic N_{10}

Shijie Liu[1, 2#], Lei Zhao[3#], Mingguang Yao[2], Maosheng Miao[3,4*], Bingbing Liu[2*]

[1] State Key Laboratory of Superhard Materials, Jilin University, Changchun 130012, China.

[2] School of Physics and Engineering, and Henan Key Laboratory of Photoelectric Energy Storage Materials and Applications, Henan University of Science and Technology, Luoyang 471003, China.

[3] Department of Chemistry and Biochemistry, California State University-Northridge, Northridge, California 91330, United States.

[4] Beijing Computational Science Research Center, Beijing, 10084, China.

[#] Authors' contributions to this article are equal.

[*] To whom correspondence may be addressed. E-mail: liubb@jlu.edu.cn and mmiao@csun.edu.

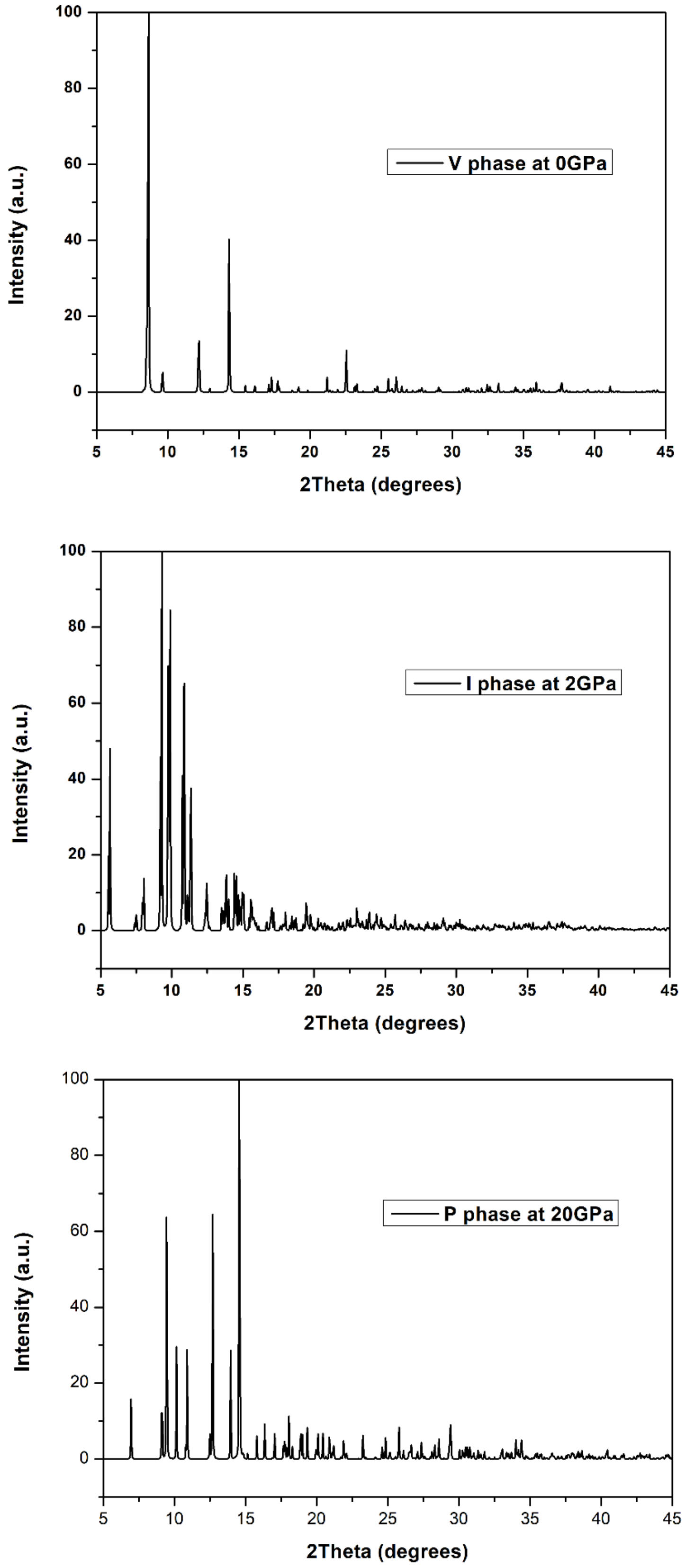

Figure S1. Calculated XRD (λ = 0.6199 Å) of V phase, I phase and P phase at 0, 2 and 20GPa, respectively.

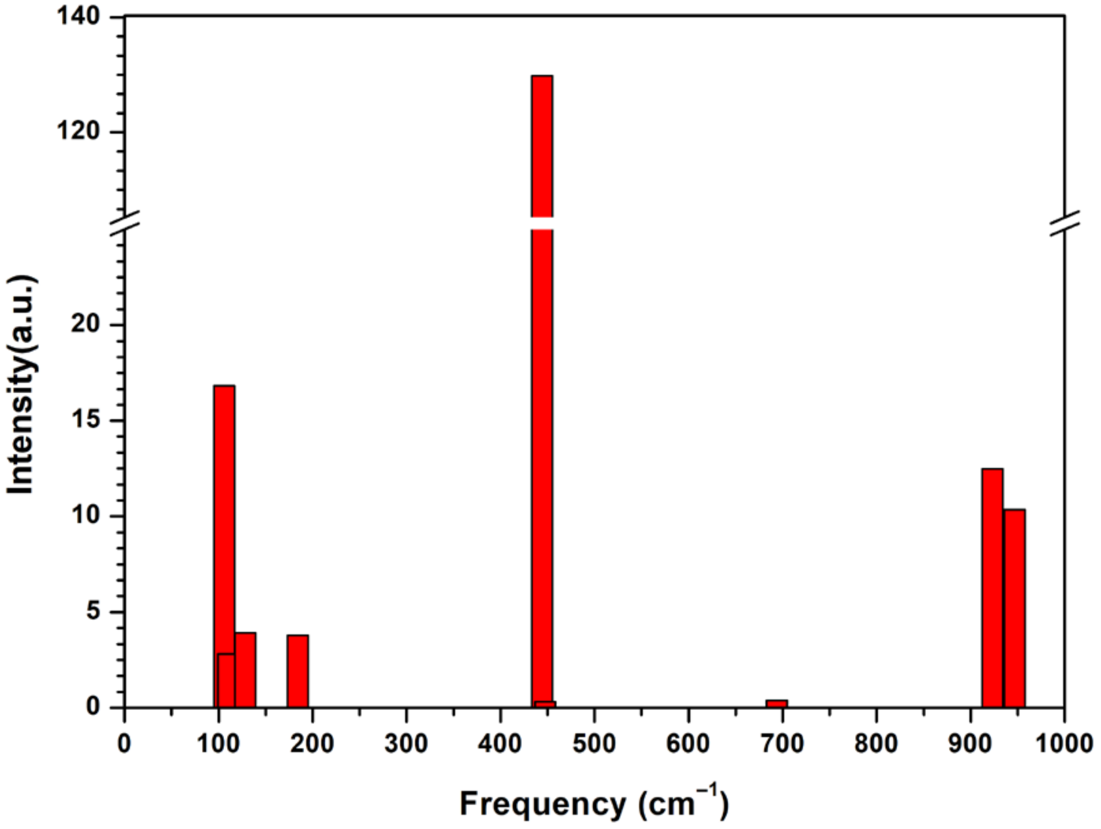

Figure S2. Calculated Raman spectrum of bispentazole molecular crystal.

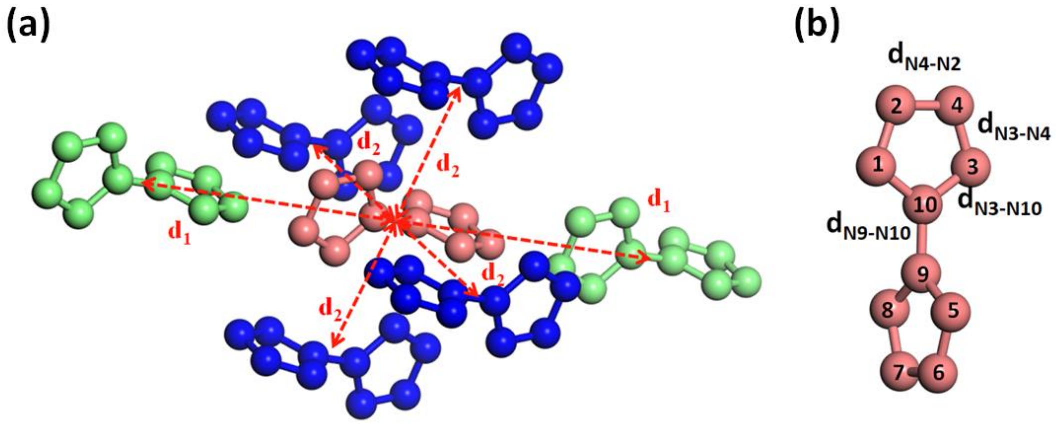

Figure S3. a) Pressure stabilizing N_{10} molecular crystal at equilibrium state; b) The magnified view of the target molecule (marked pink). Intermolecular distances (d_1 and d_2) and lengths of N-N bond in N_{10} molecule under different pressure as shown in Table S2.

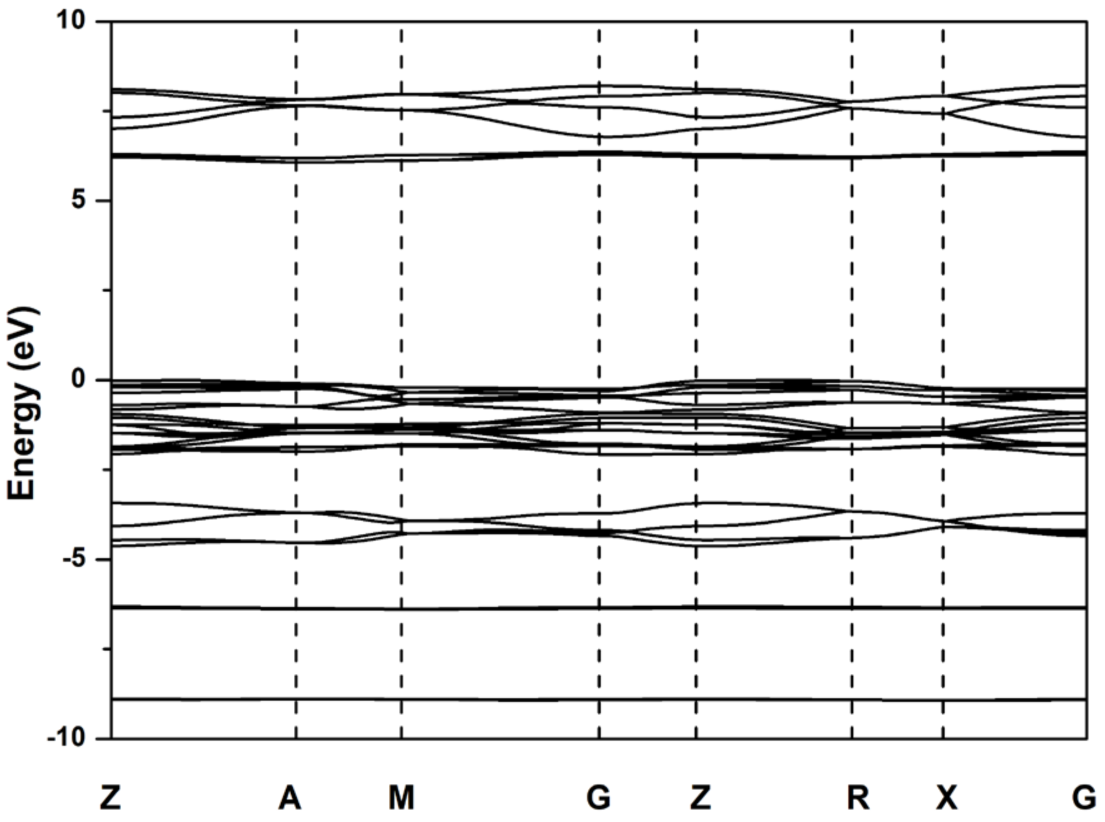

Figure S4. Band structure of the V phase at ambient pressure at HSE level.

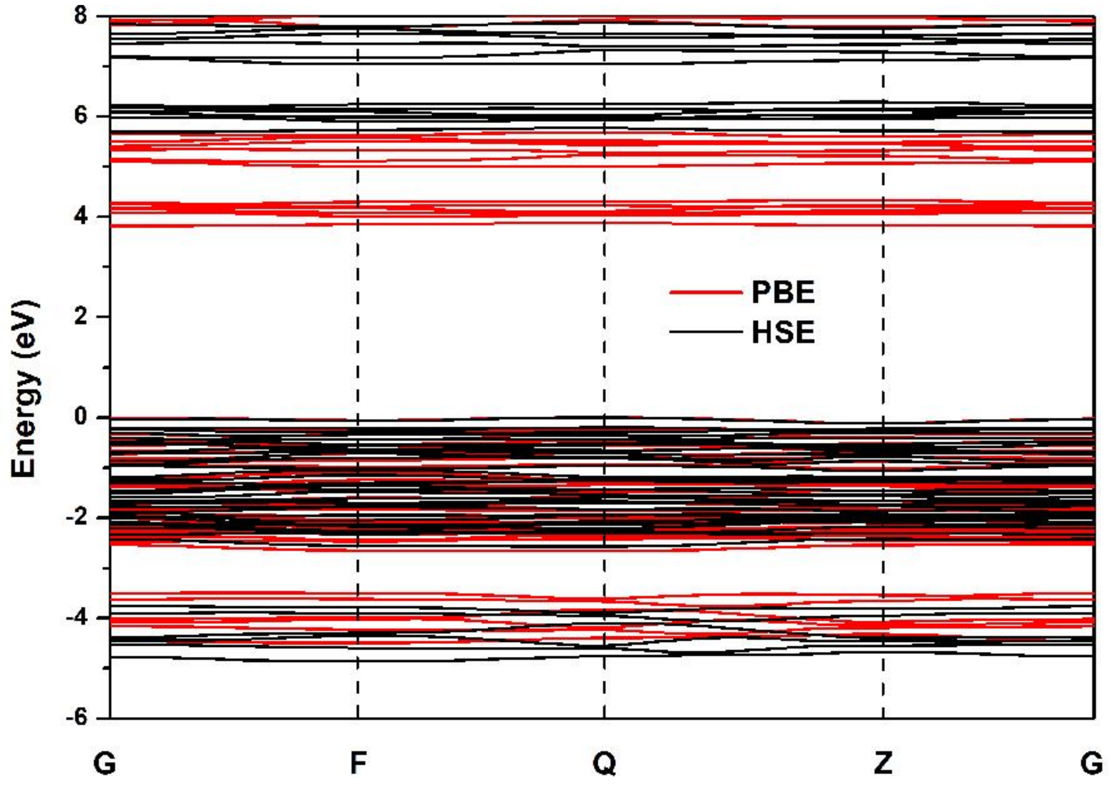

Figure S5. Band structure of the I phase at 2GPa at PBE and HSE level.

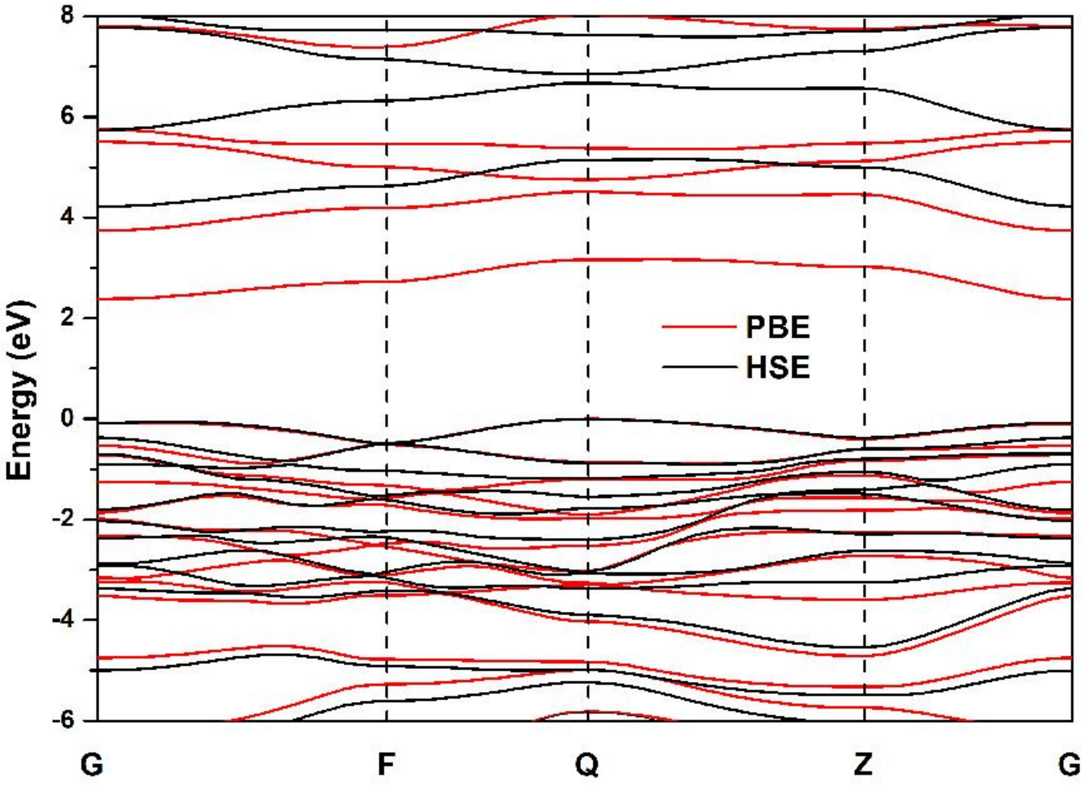

Figure S6. Band structure of the V phase at 20GPa at PBE and HSE level.

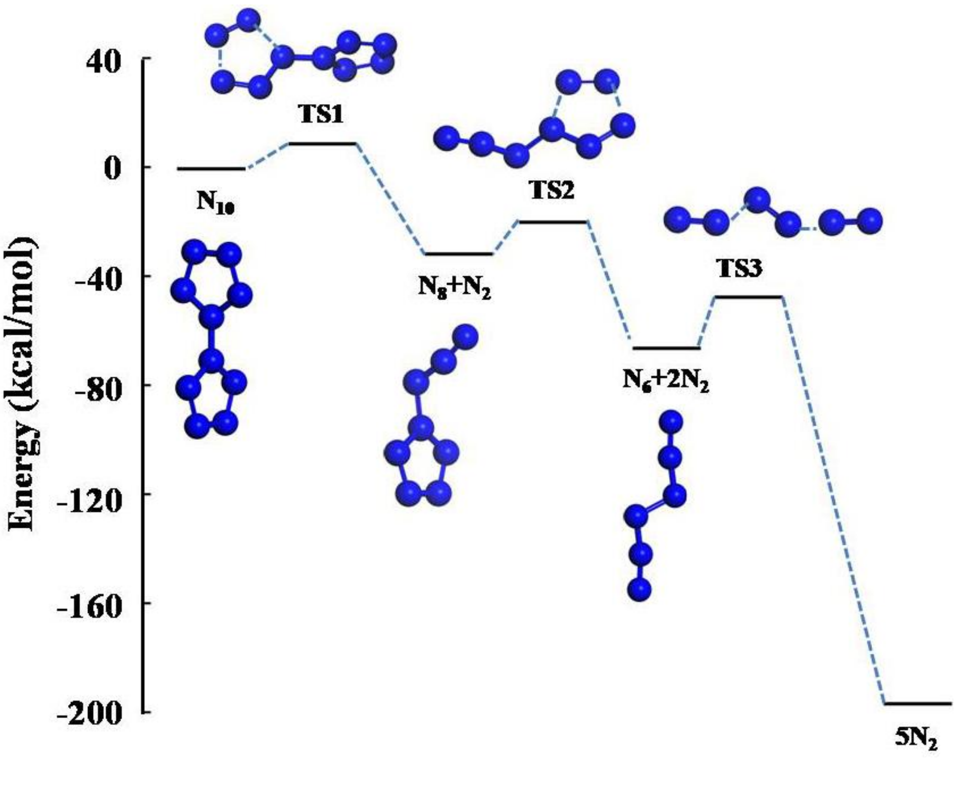

Figure S7. The calculated relevant energies (kcal/mol) and the decomposition pathway of N_{10} molecular.

Table S1. The lattice parameters of the P4/nbm (V phase), P1 (I phase) and P-1 (P phase) structures at 0GPa, 2GPa and 20GPa, respectively.

Space group	Lattice parameters (Å, °)	Wyckoff	Atomic coordinates (fractional)		
P4/nbm	a=5.8265	4h	0.50000	0.00000	0.08111
(V phase)	b=5.8265	8m	0.58338	0.91662	0.31669
	c=8.3410		0.86431	0.36431	0.17074
P1	a=7.8406	1a	0.89637	0.21970	0.30193
(I phase)	b=6.2742		0.54376	0.26891	0.75294
	c=7.7246		0.12501	0.37827	0.10739
	α=89.8817		0.14622	0.62895	0.49674
	β=110.7375		0.23807	0.94830	0.58964
	γ=89.2107		0.44132	0.64400	0.18565
			0.01341	0.25536	0.98819
			0.60217	0.93055	0.25607
			0.78598	0.64238	0.92283
			0.77250	0.35403	0.30655
			0.05660	0.94164	0.50026
			0.94359	0.11436	0.46352
			0.49718	0.07784	0.95175

			0.07137	0.05825	0.05063
			0.24914	0.25798	0.24457
			0.29348	0.75459	0.58747
			0.97626	0.79479	0.81619
			0.60534	0.09916	0.85042
			0.97942	0.87998	0.97647
			0.86095	0.78733	0.04204
			0.99949	0.74450	0.44230
			0.56206	0.76002	0.32493
			0.36923	0.23275	0.91533
			0.74171	0.32951	0.46988
			0.85621	0.64754	0.78298
			0.40506	0.74191	0.02950
			0.21569	0.05868	0.21030
			0.39882	0.35070	0.79274
			0.84625	0.17991	0.56746
			0.50735	0.91814	0.07333
P-1	a= 4.0756	2i	0.90192	0.58868	0.67805
(P phase)	b= 4.3029		0.29647	0.04234	0.78522
	c= 5.3786		0.76556	0.65406	0.84391
	α=73.2447		0.01092	0.93232	0.90518

	β=102.9719	0.22456	0.82371	0.64288
	γ=111.5879			

Table S2. Calculated bond energies in N_{10} molecule (Figure S3) at ambient pressure.

Bonds	N9-N10	N3-N10	N3-N4	N2-N4
Bond Energy (kJ/mol)	422.47	406.79	634.47	437.01

Table S3. Calculated intermolecular distances (d_1 and d_2), lengths of N-N bond in N_{10} molecule (Figure S3) and the SA under different pressure.

Pressure (GPa)	d_1 (Å)	d_2 (Å)	d_{N9-N10} (Å)	d_{N3-N10} (Å)	d_{N3-N4} (Å)	d_{N2-N4} (Å)	SA
0.001	8.341	4.120	1.3531	1.3450	1.2914	1.3741	2.329×10^{-3}
5.0	7.944	3.799	1.3423	1.3338	1.2883	1.3689	1.883×10^{-3}
10.0	7.756	3.661	1.3338	1.3256	1.2852	1.3665	1.715×10^{-3}
15.0	7.634	3.571	1.3265	1.3189	1.2825	1.3637	1.578×10^{-3}
20.0	7.544	3.504	1.3198	1.3134	1.2798	1.3614	1.495×10^{-3}
25.0	7.471	3.446	1.3136	1.3083	1.2772	1.3591	1.431×10^{-3}
